# STudy of Antithrombotic Treatment after IntraCerebral Haemorrhage: Protocol for a randomised controlled trial

**DOI:** 10.1177/2396987320954671

**Published:** 2020-09-03

**Authors:** Kristin Tveitan Larsen, Elisabeth Forfang, Johanna Pennlert, Eva-Lotta Glader, Christina Kruuse, Per Wester, Hege Ihle-Hansen, Maria Carlsson, Eivind Berge, Rustam Al-Shahi Salman, Torgeir Bruun Wyller, Ole Morten Rønning

**Affiliations:** 1 Department of Geriatric Medicine, Oslo University Hospital, Oslo, Norway; 2 University of Oslo, Institute of Clinical Medicine, Oslo, Norway; 3 Department of Public Health and Clinical Medicine, Umeå University Hospital, Umeå, Sweden; 4 Herlev Gentofte Hospital and University of Copenhagen, Herlev, Denmark; 5 Department of Clinical Sciences, Karolinska Institute, Danderyds Hospital, Stockholm, Sweden; 6 Department of Neurology, Oslo University Hospital, Oslo, Norway; 7 Department of Neurology, Nordland Hospital Trust, Bodø, Norway; 8 Department of Clinical Medicine, UiT The Arctic University of Norway, Tromsø, Norway; 9 Centre for Clinical Brain Sciences, University of Edinburgh, Edinburgh, UK; 10 Department of Neurology, Akershus University Hospital, Lørenskog, Norway

**Keywords:** Intracerebral haemorrhage, antithrombotic treatment, secondary prevention, ischaemic events, randomised controlled trial, antiplatelet, anticoagulant, atrial fibrillation, stroke

## Abstract

**Background and aims:**

Many patients with prior intracerebral haemorrhage have indications for antithrombotic treatment with antiplatelet or anticoagulant drugs for prevention of ischaemic events, but it is uncertain whether such treatment is beneficial after intracerebral haemorrhage. STudy of Antithrombotic Treatment after IntraCerebral Haemorrhage will assess (i) the effects of long-term antithrombotic treatment on the risk of recurrent intracerebral haemorrhage and occlusive vascular events after intracerebral haemorrhage and (ii) whether imaging findings, like cerebral microbleeds, modify these effects.

**Methods:**

STudy of Antithrombotic Treatment after IntraCerebral Haemorrhage is a multicentre, randomised controlled, open trial of starting versus avoiding antithrombotic treatment after non-traumatic intracerebral haemorrhage, in patients with an indication for antithrombotic treatment. Participants with vascular disease as an indication for antiplatelet treatment are randomly allocated to antiplatelet treatment or no antithrombotic treatment. Participants with atrial fibrillation as an indication for anticoagulant treatment are randomly allocated to anticoagulant treatment or no anticoagulant treatment. Cerebral CT or MRI is performed before randomisation. Duration of follow-up is at least two years. The primary outcome is recurrent intracerebral haemorrhage. Secondary outcomes include occlusive vascular events and death. Assessment of clinical outcomes is performed blinded to treatment allocation. Target recruitment is 500 participants.

**Trial status:** Recruitment to STudy of Antithrombotic Treatment after IntraCerebral Haemorrhage is on-going. On 30 April 2020, 44 participants had been enrolled in 31 participating hospitals. An individual patient–data meta-analysis is planned with similar randomised trials.

## Background and aims

Antithrombotic treatment is well-established for patients without prior intracerebral haemorrhage (ICH): antiplatelet drugs for the prevention of serious vascular events in patients with vascular disease^1,2^ and anticoagulant drugs to prevent systemic embolism in patients with atrial fibrillation,^3,4^ among other indications. However, antithrombotic drugs increase the risk of bleeding and ICH is the most severe and feared complication. Forty percent of patients suffering from ICH die within the first month and more than half the survivors become dependent on help from others.^5^

The annual risk of ICH recurrence is estimated to be 1.8–7.4%,^6^ but in the long term, these patients are at even higher risk of ischaemic events like myocardial infarction and ischaemic stroke.^7,8^ A substantial proportion of patients presenting with ICH are on antithrombotic treatment: a quarter use anticoagulant drugs,^9,10^ and more than one-third use antiplatelet drugs.^11,12^ Overall, 40–50% use, or have an indication for, antithrombotic treatment.^8,13^ After the acute phase of the ICH, the physician must decide whether to resume the antithrombotic drug or not. However, preventing a possibly devastating ischaemic event with a drug that might cause a new ICH creates a clinical dilemma because the safety of antithrombotic drugs is unknown in patients with prior ICH. Guidelines do not make clear recommendations about this,^14–16^ both policies occur in standard clinical practice,^17–19^ and clinical equipoise is demonstrated in surveys in the UK and Scandinavia.^20^

Until recently, no randomised controlled trials have assessed effects of long-term antithrombotic treatment after ICH.^21^ Observational studies have investigated the safety of antiplatelet^18,22–28^ and anticoagulant^8,29–38^ treatment after different types of intracranial haemorrhage including ICH. Overall, in these studies, antithrombotic drugs were not associated with an increased risk of recurrent ICH. However, associations with the risk of ischaemic events varied, most of the studies were small, and they were prone to selection bias and confounding by indication.

Recently, the RESTART randomised controlled trial of 537 patients investigated effects of antiplatelet treatment after ICH.^39^ During a median follow-up period of two years, RESTART did not show an increase in the rate of recurrent ICH from antiplatelet drugs, but on the contrary a non-significant reduction in the risk of recurrent ICH (adjusted hazard ratio 0.51, 95% CI 0.25–1.03; *p* = 0.060). The RESTART results are reassuring for antiplatelet treatment but need to be confirmed by other randomised trials.

The recently presented NASPAF-ICH trial (Non-Vitamin K Antagonist Oral Anticoagulants for Stroke Prevention in Patients With Atrial Fibrillation and Previous Intracerebral Hemorrhage Study) randomised 30 participants with atrial fibrillation and previous ICH to Non-Vitamin K antagonist oral anticoagulants versus Aspirin.^40^ The primary feasibility outcome was recruitment rate, which was 3.1 participants/site/year. During a mean follow-up period of 1.53 years, no recurrent ICH occurred. However, this was a phase II feasibility trial, too small to draw conclusions about clinical outcome data. More evidence is needed to guide both anticoagulant and antiplatelet treatment after ICH.

It is also uncertain whether one should give or avoid antithrombotic treatment in ICH patients with many cerebral microbleeds or cerebral amyloid angiopathy (CAA), who at the same time have a high risk of ischaemic events. Cerebral microbleeds are associated with increased risk of ICH,^41–43^ but also with increased risk of ischaemic stroke.^41,44^ Lobar microbleeds might indicate CAA, which is also associated with a higher ICH recurrence rate.^45^ However, in a cohort study of 1012 patients with atrial fibrillation and prior ICH, even those who fulfilled criteria for possible or probable CAA showed an association between resuming anticoagulation and better outcome on mortality and functional status.^36^ The interaction between these imaging findings and the effects of antithrombotic treatment is still unknown.

The primary aim of STudy of Antithrombotic Treatment after IntraCerebral Haemorrhage (STATICH) is to assess the effects of antithrombotic drugs on the risk of recurrent ICH and occlusive vascular events after ICH. The secondary aim is to assess whether brain imaging findings, like cerebral microbleeds, modify the effects of antithrombotic treatment after ICH.

## Methods

### Trial design and overview

STATICH is a Scandinavian, investigator-led, multicentre, randomised controlled, open trial of antithrombotic treatment for prevention of ischaemic disease in patients with prior ICH. The trial is conducted according to Good Clinical Practice and The Declaration of Helsinki. Regulatory agencies and ethics committees in the three participating countries have approved the trial. The EudraCT number is 2014–002636-13 and the ClinicalTrials.gov number is NCT03186729.

### Patient population and consent

Participants are recruited during hospital admission or in an outpatient clinic. Patients are eligible for the trial if they are 18 years old or more, have had a prior non-traumatic ICH minimum one day ago and have an indication for antithrombotic treatment for prevention of ischaemic events. There must be no preceding traumatic brain injury or underlying structural cause of the ICH, defined as tumour, aneurysm, vascular malformation, intracerebral venous thrombosis or haemorrhagic transformation of an ischaemic stroke. There must be no compelling indication for antithrombotic treatment (e.g. recent coronary artery stenting or prosthetic metallic heart valve). Detailed eligibility criteria are shown in Table 1.

**Table 1.** Eligibility criteria.

Inclusion criteria
• Patient age ≥18 years
• Non-traumatic, primary intracerebral haemorrhage ≥24 hours ago, and:
• No preceding traumatic brain injury, based on history from the patient/witness of spontaneous symptom onset, and brain imaging appearances consistent of spontaneous intracerebral haemorrhage (i.e. any brain/bone/soft tissue appearances of trauma must have occurred secondary to a spontaneous intracerebral haemorrhage)
• No underlying structural cause (e.g. aneurysm, tumour, arteriovenous malformation, intracerebral venous thrombosis, or haemorrhagic transformation of an ischaemic stroke)
• Patient has indication for antithrombotic (i.e. anticoagulant or antiplatelet) drug for the prevention of ischaemic events, either antiplatelet drugs for patients with vascular disease, or anticoagulant drug for patients with atrial fibrillation. Indication for antiplatelet drugs can be previous ischaemic stroke, myocardial infarction, other occlusive arterial disease, or arterial stents or other arterial implants (secondary prevention), or patients with known significant atherosclerotic arterial disease, such as carotid or coronary artery stenosis or mobile aortic atheroma (primary prevention)
• Consent to randomisation from the patient (or personal/legal/professional representative, if the patient does not have mental capacity to give consent, and waiver of consent is acceptable in the patient’s country)
• CT and/or MRI is performed before randomisation
Exclusion criteria
• Clear indication for antiplatelet or anticoagulant treatment (e.g. recent coronary artery stenting, or prosthetic metallic heart valves)
• Contraindications to the antithrombotic drug that will be administered
• Patient is pregnant, breastfeeding or of childbearing potential and not using contraception. A woman of childbearing potential must undergo a pregnancy test before randomisation and the result must be recorded in the case report form. Women of childbearing potential randomised to active treatment must use effective methods of contraception and undergo regular pregnancy testing during follow-up, and the results must be recorded in the case report forms.
• For MRI examination: Contraindication to the brain MRI
• Malignancy with life expectancy less than two years

Written informed consent is obtained from the participant by the treating physician before enrolment. If the participant lacks capacity to consent, consent is obtained from the participant’s legal representative, if this is accepted in the respective country. If such a participant regains capacity to consent a later stage, written informed consent is then obtained from the participant.

### Imaging

All participants have cerebral MRI or CT scans performed in relation to the qualifying ICH, but before randomisation, if there are no contraindications. For the MRI scan, susceptibility-weighted imaging sequence(s), T1, T2 and FLAIR images in a 3 T MRI scanner are preferred, but a standard MRI stroke protocol in a 1.5 T MRI scanner is acceptable. All cerebral CT and MRI scans performed after the qualifying event and before the time of randomisation are sent on CD ROM to the Trial Co-ordinating Centre in DICOM viewer format. In selected hospitals, participants will undergo a new cerebral MRI scan after two years, to enable assessment of how antithrombotic treatment affects the development of different imaging findings. Two radiologists, blinded to treatment allocation, will independently interpret the CT and MRI scans according to pre-specified criteria.

### Randomisation and intervention

**Figure 1.**
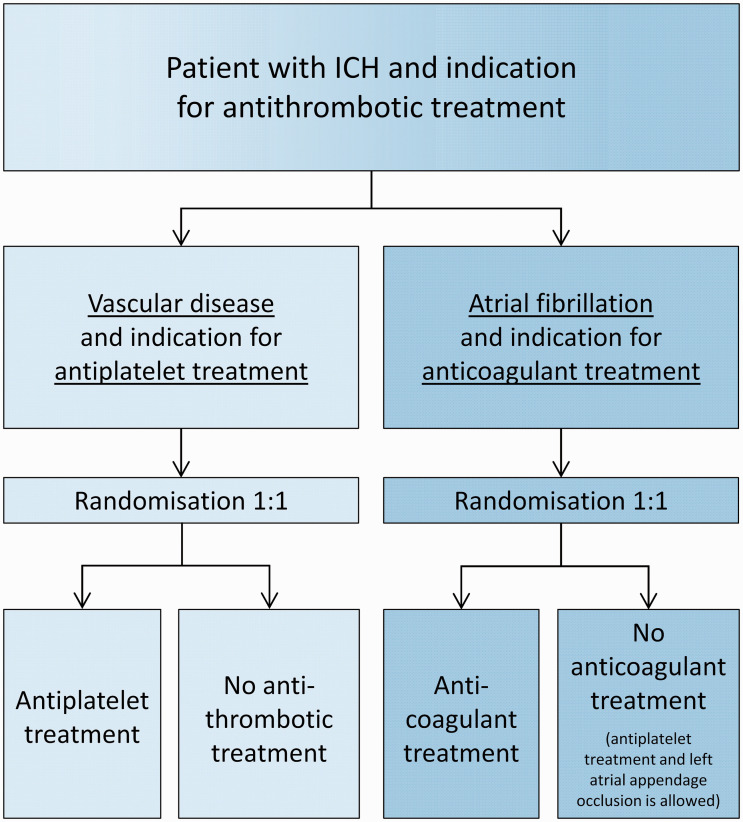
STATICH flowchart.

The intervention is treatment with an antithrombotic drug. The control is a policy of avoiding these drugs. Participants with vascular disease and indication for antiplatelet treatment are randomised to starting antiplatelet treatment or avoiding antithrombotic treatment altogether. Participants with atrial fibrillation and an indication for anticoagulant treatment are randomised to starting anticoagulant treatment or avoiding anticoagulant treatment (which may include antiplatelet drugs or left atrial appendage occlusion; Figure 1, flowchart).

Randomisation is performed by a web-based, central randomisation system that allocates the participants to intervention or control, according to a minimisation algorithm without a pre-determined sequence. The algorithm prevents predictable alternation of allocation by randomly allocating the first participant with a probability of 0.5 to one of the arms. Adaptive stratification is used to allocate each subsequent participant with a probability of 0.8 to the arm that minimises the difference between the arms, with respect to baseline characteristics in the previously randomised participants. Treatment allocation for an individual participant is revealed only after the baseline data are submitted, and is open to participants, treating physicians and local research staff, as well as personnel in the Trial Co-ordinating Centre assessing protocol adherence. The dedicated personnel recording clinical outcome events are blinded to treatment allocation. The central Event Adjudication Committee that evaluates all outcome events is also blinded to treatment allocation.

The timing of randomisation after the qualifying ICH is up to the treating physician. If the participant is allocated to an antithrombotic drug, the drug should be initiated within 24 h after randomisation. For participants allocated to treatment, the treating physician is responsible for choosing the type and dose of the antithrombotic drug, in accordance with standard clinical practice.

### Follow-up

Participants receive a patient card containing the study code, the name of the Sponsor and the telephone number to the Trial Co-ordinating Centre. When a participant is included, and thereafter at six months’ intervals, the national Co-ordinating Centre sends a letter to the participant and the general practitioner to remind them of the treatment allocation. The participant is contacted by the national Co-ordinating Centre at one month and every six months for at least two years after randomisation by telephone. The focus is the participant’s safety, any adverse events and adherence to the protocol. Recording of adherence and outcomes is done by structured interviews with the participant, his/her nearest relative and/or the general practitioner, and by collecting data from medical records. If a participant develops any exclusion criteria, withdrawal from the allocated treatment may be considered. The follow-up schedule continues for all randomised participants, unless they choose to withdraw from follow-up. The pre-determined duration of follow-up for the primary/secondary outcomes is two years.

Follow-up at 5 and 10 years will occur by telephone interviews with participants, collection of data from medical records and linkage with participants’ data in national registers, to determine long-term survival and risk of vascular events.

Co-enrolment into other trials during follow-up is allowed by the chief investigator, if no interaction between trial interventions can be expected, and co-enrolment does not interfere with follow-up in STATICH.

### Assessment of outcome events

The primary outcome is occurrence of new, symptomatic ICH over at least two years of follow-up. Secondary outcomes are other intracranial haemorrhagic events, major and minor extracranial haemorrhagic events, occlusive vascular events (transient ischaemic attack, ischaemic stroke, unstable angina, acute myocardial infarction, peripheral arterial occlusion, mesenteric ischaemia, central retinal arterial occlusion, revascularisation procedures (carotid, coronary, peripheral arterial), symptomatic deep vein thrombosis and symptomatic pulmonary embolism), vascular death, all-cause death and functional status according to the modified Rankin Scale (Table 2). Data collection forms used in the trial are available from the corresponding author upon request.

**Table 2.** Outcome events.

Primary outcome
Fatal or non-fatal symptomatic intracerebral haemorrhage (neurological deterioration or death associated with intracerebral haemorrhage found on CT scan, MRI scan, or by autopsy) over at least two years of follow-up
Secondary outcomes
• Occlusive vascular events: transient ischaemic attack (requiring hospitalisation), ischaemic stroke, unstable angina (requiring revascularisation), acute myocardial infarction, peripheral arterial occlusion, mesenteric ischaemia, central retinal arterial occlusion, revascularisation procedures (carotid, coronary, peripheral arterial) symptomatic deep vein thrombosis or symptomatic pulmonary embolism
• Symptomatic (spontaneous or traumatic) epidural, subdural or subarachnoid haemorrhage
• Major extracranial bleeding, as defined by the International Society on Thrombosis and Haemostasis (ISTH-criteria^50^):
1. Fatal bleeding, and/or
2. Symptomatic bleeding in a critical area or organ, such as intracranial, intraspinal, intraocular, retroperitoneal, intra-articular or pericardial or intramuscular with compartment syndrome, and/or
3. Bleeding causing a fall in haemoglobin level of 20 g/L (1.24 mmol/L) or more, or leading to transfusion of two or more units of whole blood or red cells.
• Clinically relevant non-major bleeding (ISTH-criteria^51^):
1. Requiring medical intervention by a healthcare professional, or
2. Leading to hospitalisation or increased level of care, or
3. Prompting a face to face (i.e. not just a telephone or electronic communication) evaluation
• Vascular death
• Death from any cause
• Functional status (according to the modified Rankin Scale) at two years

### Subgroup analyses

Relevant subgroups are those defined by age, type of antithrombotic drug, timing of treatment initiation and haemorrhage location. For participants randomised to anticoagulants versus control, relevant subgroups also include CHA_2_DS_2_VASc score^46^ and HAS-BLED score.^47^ Pre-specified subgroup analyses will also examine possible interactions between imaging findings, like cerebral microbleeds, and effects of antithrombotic treatment on the risk of recurrent ICH.

### Statistical considerations

The risk of ICH recurrence is estimated to be 1.8–7.4% per year.^6^ There is uncertainty about the relative increase in the risk of recurrent ICH on antiplatelet or anticoagulant drugs, but observational studies indicate no increased risk of recurrent haemorrhage compared to not taking antithrombotic drugs.^8,18,22–38^ A fourfold increase in the risk of recurrent ICH on antithrombotic drugs (from about 2% to 8%) would be considered unacceptable and higher than any plausible effect antithrombotic drugs would have on ischaemic events. With 500 participants randomised to antiplatelets versus control, or to anticoagulants versus control, STATICH will have more than 80% power at the 5% significance level to detect such a difference. STATICH aims to randomise 500 participants to antiplatelets versus control, and more than 50 participants to anticoagulants versus control. Information from the latter will be used to assess the plausibility of a net benefit in a larger main study. These sample size calculations were made before results from the RESTART trial were reported and are therefore based on prior assumptions. The target sample size and inclusion period will be reviewed and adjusted by the Trial Steering Committee based on overall data (not by treatment group) on primary outcome events, the number of participants in pre-specified subgroups and completeness of follow-up.

The primary analysis will be restricted to the primary outcome and performed separately for the antiplatelet and anticoagulant part of the trial. Subgroup analyses will be performed for the primary outcome and interactions will be tested, if appropriate. Secondary analyses will be performed for the secondary outcomes. All participants will be included in the analyses. To retain the benefit of randomisation, all participants will be analysed according to the ‘intention to treat’ principle, i.e. in the group to which they were allocated, irrespective of whether they adhere to the allocated treatment. We intend to publish a separate statistical analysis plan before the follow-up is complete and the data base is locked.

### Safety

STATICH is a pragmatic trial, investigating which of two forms of standard clinical care is most beneficial. The drugs are well-known and used for standard indications. Data on outcomes and serious adverse events are routinely collected and evaluated. Procedures for management of adverse drug reactions are specified in the standard operating procedures. In case of an unexpected serious adverse event (SUSAR), the Trial Co-ordinating Centre should be notified immediately. Sponsor will report the SUSAR in an expedited manner to the authorities according to the applicable regulatory requirements. The Data Monitoring Committee will perform un-blinded reviews of outcome events in all participants and in the pre-specified subgroups during the trial. The committee will also perform an un-blinded interim analysis of the primary outcome variable when half the participants have been included.

### Data quality

The trial will be monitored according to the risk assessment. Monitoring will be independent from investigators and Sponsor. The central randomisation system checks eligibility for all participants. To facilitate protocol adherence and fulfilling of the trial’s objectives, there will be frequent telephone contacts between the Trial Co-ordinating Centre and participating hospitals.

### Study organisation

Oslo University Hospital is the Sponsor. The Director of Research acts as the Sponsor’s legal representative. The Trial Co-ordinating Centre is hosted within the Stroke Research Group at Oslo University Hospital (Ullevål). The Trial Co-ordinating Centre works in close collaboration with the Trial Imaging Centre at Akershus University Hospital, and with National Co-ordinating Centres in Sweden and Denmark. The STATICH Study Group comprises all hospitals participating in the trial. A list of all participating hospitals is available from the corresponding author on request.

### Publication policy

The primary results of the trial will be published in international peer reviewed journals, presented at international scientific meetings, and communicated as popular science articles in public media.

### Current trial status

The current protocol version is dated 05 March 2020. Important protocol modifications are communicated to relevant parties during the trial. Recruitment started in June 2018 and is on-going. On 30 April 2020, a total of 44 participants had been randomised in 31 participating hospitals: 21 to antiplatelets versus control, and 23 to anticoagulants versus control.

## Discussion

Randomised controlled trials assessing effects of long-term antithrombotic treatment after ICH are needed for several reasons. First, it is likely that antithrombotic treatment prevents ischaemic events even after ICH, but these patients were not included in previous randomised trials of antithrombotic treatment, and the effect of these drugs on the risk of recurrent ICH is uncertain. Second, although observational studies have not shown an association between antithrombotic treatment and increased risk of recurrent ICH,^8,18,22–38^ observational studies are insufficient to guide this treatment decision. Third, guidelines vary, and there is evidence of clinical equipoise about the use of antithrombotic treatment after ICH in clinical practice. Recruitment to STATICH might be hampered by the physician’s assessment of patient frailty and short life expectancy after ICH. However, many of the long-term survivors after ICH live functionally independent lives^48^ and need optimised prevention of vascular disease.

The recently published RESTART randomised trial showed that restarting antiplatelet treatment at a median of 76 days after ICH might halve the risk of recurrent ICH.^39^ These results were opposite to prior expectations and their reproducibility should be investigated in other randomised trials. Furthermore, RESTART did not identify any harmful effects from antiplatelet treatment in the subgroups with lobar haemorrhage or cerebral microbleeds of different patterns.^49^ The subgroups were small and more data are needed, but the results permit inclusion of patients with a variety of imaging findings into other randomised trials.

In addition to RESTART and STATICH, only one other trial is currently assessing effects of antiplatelet treatment after ICH (RESTART France, NCT02966119), to our knowledge. Anticoagulant treatment after ICH is being investigated by several other on-going trials: APACHE-AF (NCT02565693), SOSTART (NCT03153150), A3ICH (NCT03243175), ENRICH-AF (NCT03950076), ASPIRE (NCT03907046) and PRESTIGE-AF (NCT03996772), in addition to the completed NASPAF-ICH feasibility trial. Data from – hopefully – all these trials will be combined with results from STATICH in a prospectively planned, individual patient–data meta-analysis. This meta-analysis aims to provide statistical power to evaluate both the overall effects of antithrombotic treatment after ICH, as well as the effects in subgroups of patients, e.g. by ICH location and other brain imaging findings.
